# Control of the Germinal Center by Follicular Regulatory T Cells During Infection

**DOI:** 10.3389/fimmu.2018.02704

**Published:** 2018-11-20

**Authors:** Brodie Miles, Elizabeth Connick

**Affiliations:** ^1^Vaccine and Gene Therapy Institute, Oregon Health & Science University, Beaverton, OR, United States; ^2^Division of Infectious Diseases, University of Arizona, Tucson, AZ, United States

**Keywords:** follicular regulatory T cells, germinal center, immune regulation, infection, vaccination

## Abstract

Follicular regulatory T cells (Tfr) are a unique subset of CD4 T cells that control and impact adaptive immune responses in the lymphoid follicles and germinal centers (GC). Since their relatively recent discovery, several studies have revealed that Tfr interact with other cells within this niche and shape ensuing responses. Recent advances defining the functional and developmental characteristics of Tfr have revealed key characteristics of Tfr differentiation, GC recruitment and retention, and regulatory properties. Further, Tfr shape the GC response and balance tolerance through interactions with Tfh, by modifying Tfh number, diversity and function, as well as with B cells. Mechanisms by which Tfr regulate the GC include cell-to-cell interactions with Tfh and B cells, as well as altering their environment through cytokine production and sequestration. Tfr have been shown to have a diverse T cell receptor (TCR) repertoire and can be specific for immunizing agents, demonstrating a potential role in vaccine development. Due to these important characteristics and functions, Tfr play a major role in immune tolerance, response to infection, and vaccine efficacy.

## Characteristics and functions of Tfr

Follicular regulatory T cells (Tfr) are a subset of regulatory T cells (Treg) that suppress follicular T helper cells (Tfh) and B cells. Tfr were first identified in mice and shown to play a crucial role in the germinal center (GC) response and antibody production (1–3). Tfr express high levels of CXCR5, which directs them to follicles and GCs (3). Similar to Tfh, Tfr require ICOS and CD28 to promote development and maintenance (2), and are inhibited by high levels of PD-1 (4) and IL-2 (5). Tfr require T cell co-stimulation for growth and development, as CD28 deletion results in nearly total loss of Tfr in spleen and lymph node after immunization (2).

Tfr express high levels of the transcription factor Bcl-6 (1). Uniquely, Tfr are able to co-express the transcription factors Bcl-6 and Blimp-1, although these factors are typically believed to be part of a negative feedback loop (1, 3). Loss of Bcl-6 results in almost a complete loss of Tfr, however, almost paradoxically, Blimp1 is the master regulator of Tfr function (2). Further, NFAT-2 is a critical Tfr transcription factor that promotes Tfr differentiation and retention in the GC and lymph node follicles through upregulation of CXCR5 expression (6). Lastly, SLAM-associated protein (SAP) is increased in fully differentiated Tfr and plays a critical role in Tfr maintenance by mediating the interactions of Tfr with B cells in the GC, as SAP deletion results in the loss of GC B cells and defective GCs (2).

Similar to Tfh, Tfr require the GC microenvironment and GC B cells for their development and function, as they are dependent on ICOS-ICOSL interactions for regulatory function and expansion. However, although phenotypically similar to Tfh, Tfr originate from CD25+ Foxp3+ Treg precursors while Tfh originate from CD4+Foxp3- T cell precursors (1, 2). Antigen presenting cells play a definitive role in development and maturation of Tfr. B cells in the GC of lymph node follicles promote optimal Tfr growth and differentiation. In addition, dendritic cell (DC) subsets in mucosal and peripheral tissue sites promote Tfr development. Mice that were depleted of DCs had a significant reduction in the proportion of Tfr (7). This suggests that Tfr development and maintenance can be highly tissue specific and require multiple cell subsets and environmental cues. In addition, Tfr express potentially autoreactive TCR repertoires leading to their ability to suppress autoimmunity, in contrast to Tfh, which arise from antigen-responsive TCRs allowing for promotion of antibody responses (8).

The GC microenvironment is important to driving follicular T cell differentiation while maintaining cell phenotype and function. After their differentiation and antigen imprinting, Tfr either enter circulation to become memory Tfr or localize to the B cell zone to interact with B cells and Tfh. Studies in mice have demonstrated that memory Tfh lose expression of CXCR5, PD-1, and Bcl-6 after adoptive transfer into naïve hosts (9). However, circulating Tfh from human donors have been cultured up to 3 weeks without stimulation and maintained CXCR5 and PD-1 expression (10). Importantly, Tfr were included within the Tfh subset in both of these analyses. Factors that promote Tfr egress from the follicle are unknown. Receptor mediated endocytosis of CXCR5 has been described for B cells (11). It is possible that CXCR5 is downregulated transiently on T cells in the context of high levels of CXCL13 in the follicle, thereby enabling these cells to exit the follicle before CXCR5 is re-expressed. In addition, Tfr may upregulate other receptors that promote migration to other sites. For example, in Sage et al.'s study (7), peripheral blood Tfr were enriched for CXCR3, which may direct Tfr to other sites outside of the lymph nodes. When Tfr migrate out of the follicle, they have similar CXCR5 levels, but diminished ICOS expression and PD-1 expression, indicating the importance of the GC microenvironment for development and maintenance of Tfr phenotype (7). Adding more complexity, a recent study characterized a subset of Tfr lacking CD25 expression, thus being independent of IL-2 signaling and allowing for increased CXCR5 and Bcl-6 expression (12).

## Tfr regulation of Tfh

Similar to Treg, Tfr are able to suppress immune responses through a variety of mechanisms. The suppressive function of Tfr was initially shown in mouse adoptive transfer experiments where fully differentiated Tfr were shown to potently suppress antigen-specific antibody production and GC responses (2). Further, isolated Tfr cultured with GC B cells from immunized mice showed that Tfr substantially diminish IgG output *in vitro* (4). Similar to Treg, Tfr utilize mechanisms to suppress Tfh and B cells (described below) such as downregulation of co-stimulatory molecules, cytokine production, and direct physical disruption, while metabolic disruption and cytolytic functions remain mostly unexplored.

One of the key regulatory effector molecules of Tfr is CTLA-4. CTLA-4 has been shown to control Foxp3+ Treg functions and act as a co-inhibitory molecule to dampen immune responses by preventing CD28-B7 co-stimulatory interactions (13, 14). Through genetic deletions in mouse models, CTLA-4 expression by Tfr has been shown to play a crucial role in Tfh differentiation and functional responses. CTLA-4 expression on Tfr potently suppresses Tfh generation, differentiation, and subsequent B cell responses (15, 16). CTLA-4 expression in Treg controls Tfh antigen-specific expansion and Tfh cell numbers (16). It should be noted, however, that global deletion of CTLA-4 altered Tfh numbers rather than simply Tfr CTLA-4 levels (15). Blockade of CTLA-4 resulted in spontaneous Tfh differentiation and large GC expansion in a CD28-dependent manner, as CD28 heterozygosity also reduced Tfh differentiation while leaving other facets of T cell activation unaltered (17).

Mouse models have also demonstrated that expression of PD-1 plays a large role in the function of Tfr, as similar to most cell types engagement of PD-1 leads to loss of effector function and exhaustion. PD-1 expression on Tfr markedly reduced their ability to suppress Tfh function, while PD-1 deficiency led to heightened suppressive ability (4). In a study using adoptive transfer of OT-II cells into Cd3e-deficient mice (i.e., mice with abnormally low levels of lymphocytes in the blood), which causes increases of Tfh and impaired GC responses, the addition of Treg restored normal Tfh cell numbers, B cell distribution within the GC, and somatic hypermutation rates (18). Further, PD-L1 deficient mice have higher percentages of Tfr and increased Blimp-1 and Bcl-6 expression, demonstrating that PD-1 signals could inhibit Tfr differentiation and accumulation (4). Tfr responses can also be manipulated by Tfh function, as IL-21 serves as negative feedback for downregulation of CD25 (and IL-2 responsiveness) through Bcl-6 expression (19). Human Tfr reduce IL-21 and IL-4 production by Tfh in an *ex vivo* HIV infection model by a mechanism that is contact dependent (20). Thus, Tfr may regulate Tfh production of IL-21 both to limit the GC response and also to prevent loss of their own effector functions.

The dynamics of Tfr and Tfh interactions can vary based on the microenvironment and circumstances of immune responses. Tfr were shown to accumulate in number and proportion to Tfh in untreated. chronically HIV-infected individuals' lymph nodes (20). Similarly, increases in percentages of circulating cells with a follicular regulatory phenotype are seen in individuals with untreated chronic hepatitis B infection (21, 22). In an *ex vivo* HIV infection model, Tfr led to a decrease in Tfh ICOS expression and inhibited rates of Tfh proliferation (20). Depletion of Tfr in mice did not alter Tfh and GC B cell populations upon immunization, however, the quality of the GC response was diminished as antigen-specific antibody responses were altered and IgG production was reduced (23). Interestingly, Foxp3 depletion in mice was shown to compromise influenza-specific Tfh responses due to suppression of Tfh differentiation via increased IL-2 availability (24), thereby demonstrating a positive role for Tfr in favorable Tfh responses. Rather than absolute numbers of Tfr and Tfh, the ratio of Tfr to Tfh in the GC is thought to be critical to generating immune responses (4), as well as regulating autoimmunity (25).

Tfr have been demonstrated to allow initial B cell activation, but to physically disrupt Tfh-B cell interactions and thereby limit GC effector cell function (26). RNAseq transcriptome analysis revealed that global gene expression does not differ substantially between Tfh that have been suppressed by Tfr and (unsuppressed) active Tfh populations. Further transcriptome analysis revealed that Tfr suppressed Tfh expression of key effector molecules such as IL-4, IL-21, IL-10, and CD28, but did not alter expression of key transcription factors such as Bcl-6 or CXCR5 expression. This, as the authors suggest, demonstrates that Tfr suppress Tfh in a manner the allows them to retain their Tfh differentiation and maintenance profile, but inhibit upregulation of their specific effector molecules.

## Tfr regulation of B cells

Tfr were first defined as a specialized subset of Treg that suppress B cell responses (2). Whereas the suppressive effects of Tfr on Tfh function are well established, few studies have investigated whether Tfr have a direct suppressive effect on B cells. Using human tonsil cells, Lim et al. demonstrated that CD4+CD25+ cells directly inhibited B cell function and antibody responses and class switch recombination (27). Importantly, because suppression of GC B cell responses is unique to Tfr, and not mediated by CXCR5- Treg (2), presumably the activity observed by Lim et al. was mediated by Tfr. Deletion of CTLA-4 specifically in Treg in mice led to large increases in antibody production, suggesting an essential role for CTLA-4 in controlling antibody production (14). Further, another study has shown mice lacking Tfh/Tfr that receive CTLA-4 –deficient Tfh/Tfr through adoptive transfer developed Tfh expansions, increased antigen-specific antibody production, but no alterations in GC B cell numbers or co-stimulatory molecule expression (15). However, Treg-specific CTLA-4 deletion was shown to lead to decreased CD86 expression on follicular, albeit not GC, B cells (15). While this shows that Tfr function and Tfh/Tfr ratio play an essential role in GC B cell responses, it does not demonstrate a direct suppression of GC B cells by Tfr.

Regardless of whether Tfr inhibit B cells directly, or through loss of Tfh effector function, B cells which have been suppressed by Tfr display unique transcriptional profiles and altered metabolic states that are associated with inability to activate Tfh and to perform class switch recombination. IgG2b transcripts were elevated, while IgG1, IgG2a, and IgA transcripts were lowered in suppressed B cell populations, as well as a few key effector molecules, such as the cytidine deaminase AID, however, most gene signatures were intact suggesting that Tfr suppress the expression of specific effector molecules (26). More profound gene expression changes in suppressed B cells were seen in B cell metabolic pathways, such as glycolysis, and upstream mediators including mTOR. Further, suppressed B cells also displayed defects in various metabolic and anabolic processes, including suppression of glycolysis. Glycolysis is crucial for antibody production (28), and interestingly this regulation of glycolysis was found to be independent of B cell proliferation (26). Intriguingly, B cell suppression persisted even in the absence of Tfr, but was reversed by IL-21.

B cells produce the pro-inflammatory chemokines CCL3 and CCL4 during GC formation and response. Tfr respond to this chemokine production by infiltrating the GC, interacting with B cells, and suppressing numbers of self-reactive B cell clones within the GC (29). PD-L1 expression on GC B cells has been shown to inhibit Tfh function based on their high expression of PD-1 and as a result preventing Tfh from optimally stimulating antibody production (30), although this study did not address if PD-L1 similarly alters Tfr function. In mesenteric lymph nodes from individuals without known infection, Tfr were found to occupy the border of the T cell zone and B cell follicle and express relatively lower levels of PD-1 than Tfh (31). Despite appearing in the GC at low frequency compared to Tfh and expressing low levels of PD-1, Tfr were able to potently suppress antibody production *in vitro* (31). Further demonstrating Tfr localization to the GC is a critical element to GC function, in patients with the autoimmune disease Sjogrens syndrome, Tfr are excluded from ectopic GCs, thus physically separating them from GC B cells and potentially contributing to autoimmunity (32).

After immediate activation of the GC response and B cell expansion, Tfr proportionally decrease, but as responses wane and the GC reaction is resolved, Tfr return to baseline levels. Within the B cell follicle, Tfr are highly motile when receiving antigenic stimulation from B cells (33). This study also showed that blocking CTLA-4-B7 interactions leads to reduced T cell interaction, reduced dendritic cell interaction, and increased T cell proliferation including Tfr during immune priming. In mice, it is well documented that Tfh and Tfr are completely dependent on the GC for their maintenance (34). However, a study of human patients receiving organ transplants showed that treatment with rituximab, which results in loss of GC B cells (anti-CD20 antibody), did not prevent the loss of Tfr populations (35). Thus Tfr may not require an ongoing GC response for maintenance in humans. Alternatively, Tfr interactions with certain cell populations in the blood or tissues such as memory or naïve B cells, as well as follicular dendritic cells, may be able to fully support Tfr maintenance in the absence of the GC. This suggests that Tfr may also be maintained in the blood of humans in a similar fashion seen with functional Tfh populations (10).

An interesting aspect of Tfr and B cell interactions apart from immune regulation are recent studies that demonstrate Tfr promote GC B cell responses. In a mouse model using lymphocytic choriomeningitis virus (LCMV), IL-10-producing Tfr promote GC formation, dark zone morphology, and B cell FOXO1 expression (36). Tfh production of IL-9 has been shown to promote cell cycling and generation of memory-precursor B cells, however, this study did not address whether this is also influenced by either IL-9 production by Tfr or IL-9 sequestering by Tfr (37). As Tfr are believed to prevent excessive antigen-driven expansion and promote clonal selection, it would be important to understand if Tfr selectively inhibit GC B cells while helping to promote memory or plasma cell function.

## Role of Tfr in response to infection

As the master regulators of GC B cell function, Tfh function, and antibody production Tfr play a crucial role in responses to microbial infections. In mouse models of acute LCMV infection, IL-10-producing Tfr were elicited and shown to support GC responses (36). In an influenza infection model in mice, high IL-2 levels at the time of infection prevented Tfr development in a Blimp1-dependent manner (5). After resolution of infection, Treg downregulated CD25 expression, differentiated into Tfr cells, and migrated into the follicles to prevent expansion of self-reactive B cell clones (5). This is in accordance with the aforementioned study that displayed loss of CD25 on Treg promotes expression of CXCR5 and Bcl-6 leading to the development of a Tfr subset (18).

Optimal GC reactions and effector B cell responses are crucial to the resolution of microbial infections. Thus, T cell help and programming of these responses play a large role in the outcome of these responses. Optimal Tfh function is required to promote adequate B cell function that leads to diverse, high avidity, antigen-specific antibody responses. In chronically HIV-infected individuals, Tfh populations expand and provide inadequate help to B cells (7, 38). These deficiencies are manifested in defects in Tfh proliferation, ICOS expression, IL-21 production, and signaling to stimulate Ig production (30). The extent to which Tfr control these Tfh responses is a current area of great interest. IL-6 promotes Tfh function and alleviates chronic viral infection in mice, due in part to inhibiting TGF-ß-dependent generation of regulatory T cells and increased Bcl-6 expression in Tfh (39). As boosted Tfh function and potential loss of immune regulation helped to resolve chronic infection in mice, Tfr expanded and reduced the quality of Tfh effector function in chronically HIV-infected humans and SIV-infected rhesus macaques (20). Further, viral infection appeared to drive Tfr differentiation in culture (20) and Tfr were shown to be highly permissive to HIV infection (40). Thus, pathogens may have the ability to program Tfr activity and numbers and alter the GC response. These interactions could lead to inadequate GC responses independent of Tfh and B cell interactions with Tfr and result in suboptimal responses and resolution of infection.

Tfr also have complex interactions with microbiota and commensals in the gastrointestinal tract. Foxp3+ cells in the GCs of Peyer's patches have been shown to regulate gut microbiota diversity and IgA selection, which in turn leads to expansion of Foxp3+ cells in a symbiotic regulatory loop (41). During inflammatory responses in the intestinal tract, Foxp3+ cells are stabilized and have enhanced suppressive capacity (42). These Foxp3+ cells also express RORγt, as well as signature regulatory receptors such as CLTA-4 and GITR, and are central to immune tolerance and limiting inflammation in the intestinal mucosa (42).

The protein kinase mTOR, which senses and integrates environmental cues to impact cellular function during viral infections, was recently characterized in Tfr after acute LCMV infection in mice (43). mTORC1 was found to be essential for Tfr differentiation during viral infection by activating STAT-3 to promote the TCF-1/Bcl-6 transcriptional axis to launch the Tfr transcriptional program. Further, it was recently demonstrated in a Bcl-6/Foxp3-deficient mouse model that loss of Tfr led to excessive lymphocyte infiltration and antibody deposition resulting in autoimmunity (44). Even slight imbalances and decreased ratios of Tfr to Tfh correlate with autoimmunity and could potentially be used as a clinical diagnostic for excessive immune activation and inadequate GC responses (25).

## Memory Tfr and vaccine development

The extent to which Tfr have memory properties and display recall function for self and foreign antigens is still unclear. PD-L1 expression on DCs limited Tfr differentiation and subsequent immune responses, suggesting that PD-1 interactions can limit Tfr priming and memory differentiation (45). Due to the complexity of sharing properties with Treg and Tfh, it is unclear if Tfr can become antigen specific and the implications this plays in vaccine development. Tfh are clearly understood to be specific for immunizing and infectious agents (34, 38, 46), whereas Treg have TCRs skewed toward recognition of self-antigens providing their role in autoimmunity (47, 48). One study has shown that Tfr are able to develop from naïve (Foxp3-) T cells in a PD-L1-dependent manner and that these cells can be specific for various immunizing agents used in a mouse model of vaccinations (49). Thus, antigen and Tfr TCR engagement likely plays a role in Tfr memory development and maintenance and therefore could be useful in modulating responses to vaccines.

Circulating Tfr are memory-like, displaying recall responses and similar effector functions to GC Tfr, but interestingly only require DCs and not B cells for differentiation (7). In addition, circulating memory Tfr expand and increase activity in GVHD patients given daily IL-2 therapy, which promotes immune tolerance (50). As circulating Tfr and lymph node-resident Tfr have differing levels of CXCR5 and ICOS expression, these subsets may have different gene expression patterns and may have different requirements for harnessing their full vaccine potential. A recent study showed that GC B cells that proliferate and produce high affinity antibodies receive help from T cells in the light zone that promotes their DNA replication and limits the duration of the S phase by regulating replication fork progression (51). It is currently unclear whether Tfr provide help to GC B cells to promote the speed of B cell replication and activation as well as high affinity selection, or limit Tfh activity to balance T cell help to B cells. The loss of STAT-3 expression in immunized mice led to a reduction of Tfr and increased levels of specific IgG1 and IgG2b antibodies, however, Tfh levels were unaffected by loss of either STAT-3 or Tfr (52).

The generation of broadly neutralizing antibodies (bnab) is a major goal of HIV vaccine development, among other infectious diseases. Most HIV vaccine development programs aim to harness the natural conditions that favor bnab production. A higher frequency of circulating memory Tfh and PD-1-expressing Treg (presumably circulating Tfr) was detected in HIV-infected individuals who make bnabs (53). In SHIV-infected rhesus macaques, lowered Foxp3 expression correlated with increased bnab production (54). This suggests that Tfr play a negative role in antibody diversity and strongly merits investigation in development of vaccines aimed at generating bnabs. IL-21 treatments are able to override Tfr function and promote Tfh and B cell functions (26). The role of Tfr in regulating antibody affinity or diversity has yet to be fully elucidated. While Tfr have a role in suppressing high and low affinity antibody production (1), it remains to be seen if Tfr control antibody diversity through affinity maturation.

## Conclusions

Tfr are a unique subset of T helper cells that play a critical role in controlling immune responses due to their functions in the GC response. They fine tune the immune response and constantly interact with Tfh and B cell populations, thereby influencing the quantity and quality of their functions. There is still much work to do to define their mechanisms of action and role in the germinal center response. A better understanding of Tfr could lead to novel therapeutic and vaccine strategies to treat many infectious diseases.

## Author contributions

All authors listed have made a substantial, direct and intellectual contribution to the work, and approved it for publication.

### Conflict of interest statement

The authors declare that the research was conducted in the absence of any commercial or financial relationships that could be construed as a potential conflict of interest.
